# From traditional medicine to modern medicine: the importance of TCM regulatory science (TCMRS) as an emerging discipline

**DOI:** 10.1186/s13020-025-01152-8

**Published:** 2025-06-26

**Authors:** Hua Hua, Jian-Yuan Tang, Jun-Ning Zhao, Ting Wang, Jun-Hua Zhang, Jiang-Yong Yu, Chang-Ming Yang, Yan-Ling Ai, Qiao-Xian Luo

**Affiliations:** 1 Key Lab of Biological Evaluation of TCM Quality of the State Administration of Traditional Chinese Medicine, Translational Chinese Medicine Key Laboratory of Sichuan Province, Sichuan Institute for Translational Chinese Medicine, Chengdu, 610041 China; 2 Sichuan Provincial Hospital of Traditional Chinese Medicine, Affiliated Hospital of Chengdu University of Traditional Chinese Medicine, Chengdu, 611137 China; 3 National Center for Nanoscience and Technology, Beijing, 100190 China; 4 Beijing Research Institute of Chinese Medicine, Beijing University of Chinese Medicine, Beijing, China; 5 Key Laboratory of Famous Doctors and Famous Prescriptions, National Administration of Traditional Chinese Medicine, Beijing, China; 6 Key Laboratory for Research and Evaluation of Traditional Chinese Medicine, National Medical Products Administration, Beijing, China; 7 State Key Laboratory of Chinese Medicine Modernization, Tianjin University of Traditional Chinese Medicine, Tianjin, 301617 China; 8 Evidence-Based Medicine Center, Tianjin University of Traditional Chinese Medicine, Tianjin, 301617 China; 9 Department of Drug Registration (Department of Supervision and Administration of Traditional Chinese Medicine and Ethnic Minority Medicine), National Medical Products Administration, Beijing, 100038 China; 10 Center for Drug Evaluation, National Medical Products Administration, Beijing, 100076 China; 11 Chengdu University of Traditional Chinese Medicine, Chengdu, 611137 China; 12 Macau Centre for Research and Development in Chinese Medicine, State Key Laboratory of Quality Research in Chinese Medicine, Institute of Chinese Medical Sciences, University of Macau, Macao, China

**Keywords:** Traditional Chinese medicine, Traditional medicine, Modern medicine, Traditional Chinese medicine regulatory science, Drug regulation, Modernization of traditional Chinese medicine, International coordination of traditional Chinese medicine regulation

## Abstract

Traditional Chinese medicine (TCM) has become a standardized medical system through systematic development across global healthcare practices. However, concerns persist regarding the safety, efficacy and quality of traditional medicinal products. Traditional Chinese medicine regulatory science (TCMRS) has emerged as an interdisciplinary field to address these challenges. This discipline integrates multidisciplinary knowledge to develop new tools, standards and approaches for systematic evaluation of benefit-risk profiles. This approach aims to ensure the quality, safety, and efficacy of TCM products, while also supporting the development of scientifically grounded regulatory frameworks that accommodate traditional medicine’s distinctive characteristics. Through comprehensive quality management from raw material sourcing to production processes and clinical validation, developing and adopting TCMRS is entrusted to significantly strengthen its regulatory oversight. This review examines the critical scientific challenges in the modernization process of TCM, analyzes the conceptual foundations of TCMRS, evaluates its pivotal role in pharmaceutical transformation, and highlights its essential function in preserving traditional knowledge while fostering therapeutic innovation. Key challenges for TCMRS implementation include reconciling traditional epistemologies with modern pharmaceutical paradigms, standardizing complex herbal formulations, and developing rigorous evaluation protocols for decoctions and compound preparations. The integration of advanced methodologies, including systems biology, network pharmacology, artificial intelligence, and nanotechnology, into regulatory frameworks, combined with enhanced international cooperation, remains a crucial strategy for tackling global public health challenges. Future development trajectories for TCMRS will prioritize lifecycle management strategies, technology-driven innovation systems, and global knowledge-sharing initiatives, propelled by advancements in life sciences and information technology. This evolution requires careful balancing of three fundamental elements: theoretical development in traditional medicine, integration of emerging technologies, and maintenance of regulatory system stability. It is crucial to innovate the working mechanisms of the TCMRS researcher alliance and the global policy-coordination mechanism for TCM regulation, enhance the conversion of basic disciplines into regulatory applications, and support the establishment of an excellent TCM regulatory system with scientific decision-making. These efforts are essential for promoting the high-quality development of the TCM industry and boosting its international influence and presence.

## Introduction

Traditional medicine encompasses medicinal practices guided by established theoretical frameworks, including Traditional Chinese Medicine (TCM), Ayurveda, Unani, and European herbal medicines. These systems hold significant historical and cultural value, characterized by distinct epistemologies, diverse therapeutic approaches, and enduring contributions to global healthcare. The *WHO Traditional Medicine Strategy (2014–2023)* advocates for improving service quality through enhanced regulation, research, and education to ensure safety, efficacy, and equitable access. It further urges governments to leverage traditional medicine’s potential in primary care and preventive health while establishing policies that secure its legal recognition and standardized growth [1]. Since 1996, China’s Modernization of Traditional Chinese Medicine initiative has systematically strived to advance TCM through theoretical innovation, refined quality standards, optimized production technologies, and global cultural dissemination. This strategy plays an important role in elevating clinical service quality and facilitating TCM’s international integration, supporting the transition of TCM from empirical practice to evidence-based global healthcare [2]. Nevertheless, persistent challenges hinder TCM modernization, including theoretical reinterpretation, quality consistency of medicinal materials, mechanistic complexity of processing methods, and evidence-based evaluation of efficacy, safety, and risk–benefit ratios. Such challenges underscore the urgent need for regulatory frameworks and technical systems tailored to TCM’s unique attributes to ensure industrial safety and sustainable innovation.

The concept of regulatory science originated in 1970 when Dr. Alan Moghissi (U.S. Environmental Protection Agency) addressed challenges in reconciling novel scientific evidence with regulatory timelines [3]. In 1985, the Institute for Regulatory Science (RSI) was established as a non-profit organization. This marks the first instance of the ‘regulatory science’ being incorporated into an institution’s name. In 1991, the FDA officially proposed the concept of “regulatory science”, and identified it as a key discipline to promote in the twenty-first century. In 2010, the U.S. Food and Drug Administration (FDA) defined it as the development and use of new tools, standards and approaches to develop products more efficiently and to evaluate product safety, efficacy and quality more effectively [4]. In 2018, the European Medicines Agency (EMA) published the “EMA Regulatory Science to 2025: Strategic Reflection” [5]. Regulatory science has been defined as the range of scientific disciplines that are applied to the safety, efficacy and quality assessment of medicinal products, providing information for regulatory decisions throughout the entire lifecycle of a medicine. It encompasses basic and applied biomedical and social science, and contributes to developing regulatory standards and tools. In the same year, the Pharmaceuticals and Medical Devices Agency (PMDA) established the Regulatory Science Center, strategically collaborating with the review and safety departments to strengthen regulatory evaluation processes, enhance pharmaceutical services, and implement robust safety protocols.

In 2019, the National Medical Products Administration (NMPA) of China initiated the implementation of the China Drug Regulatory Science Action Plan, marking the official introduction of TCMRS in government policy documents [6–9]. In the first China’s Drug Regulatory Science Action Plan, the NMPA has named 9 key priorities, one of which was “research on the safety evaluation of TCM guided by clinical practice”. In 2021, based on the experiences of implementing the first batch of key projects, the NMPA issued the second batch of key projects in the Drug Regulatory Science Action Plan. Of these new key projects, 3 of them were related to TCM. In 2023, “The TCMRS Preliminary Construction and Translational Application” became one of the top ten TCM academic achievements of the year by the China Association of Chinese Medicine [9, 10]. As an interdisciplinary field, TCMRS synthesizes multidisciplinary knowledge to develop innovative evaluation tools, standards, and approaches aligned with the characteristics of TCM. This helps to ensure product quality, safety and efficacy while providing regulatory agencies with evidence-based decision-making frameworks, enabling the integration of TCM into modern healthcare systems and fostering industry sustainability. Furthermore, TCMRS bridges TCM knowledge with contemporary medical standards, offering scientific support for the global transformation of historically rooted traditional medicines—from local practices to internationally recognized therapies.

## Progress and challenges in TCM regulatory legislation

### Scientific progress of TCM regulatory legislation

**Fig. 1:**
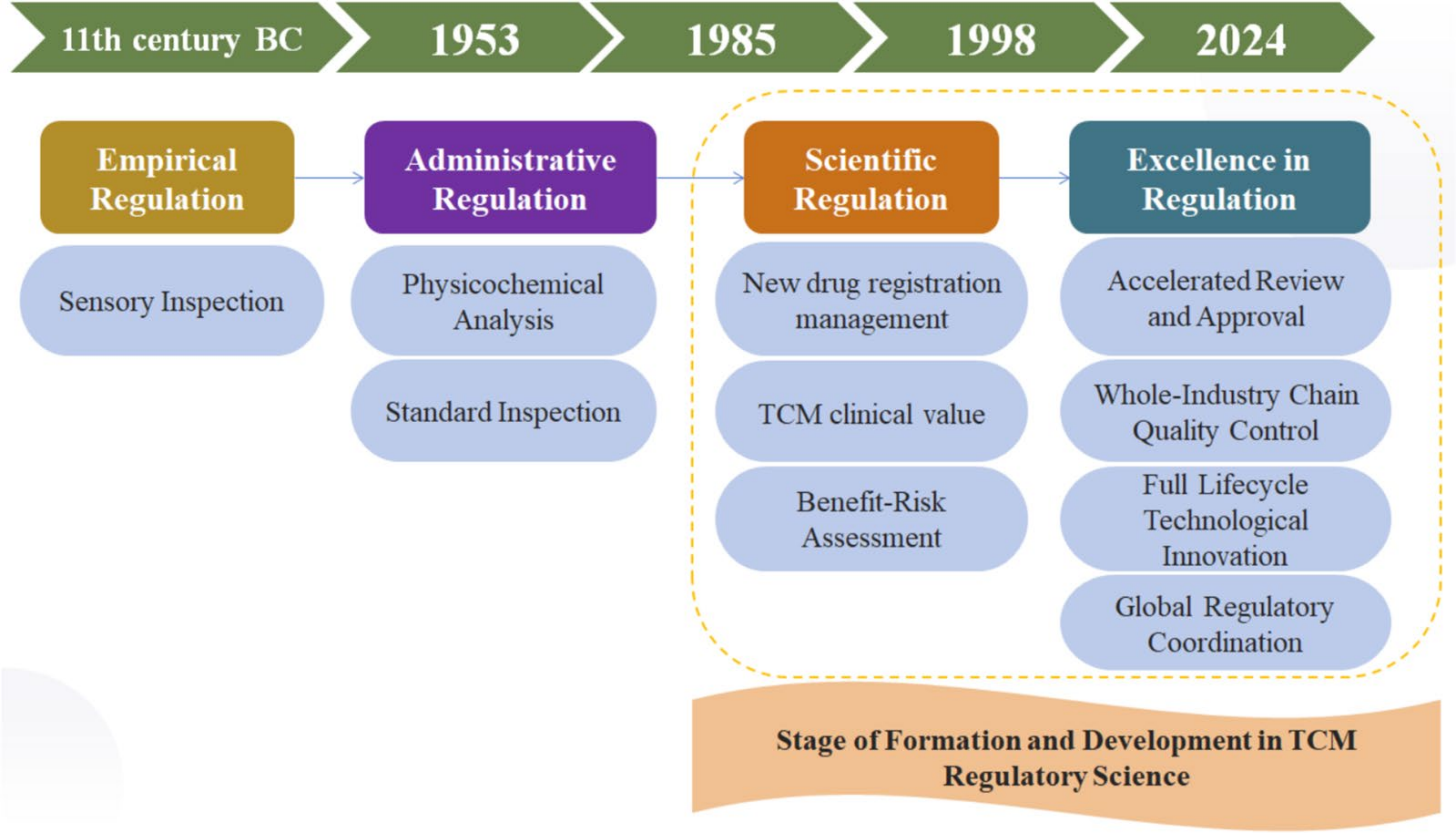
Legislative and scientific advancement of TCM regulation

China’s TCM regulatory framework traces its origins to the Western Zhou Dynasty (11th-771 BCE), with early medicinal quality management systems emerging over three millennia ago. The evolution of TCM regulation has progressed through four distinct phases: conventional TCM quality regulation (sensory trait identification); modern TCM quality regulation (physicochemical property analysis); establishment of TCM registration standards (efficacy, safety, and quality control technologies) and scientific TCM regulation (novel tools, standards, and approaches) (Fig. 1) [9]. Contemporary TCM regulation is transitioning toward a paradigm emphasizing accelerated approval processes, end-to-end quality management, lifecycle innovation, and international regulatory harmonization.

Historically, TCM quality regulation relied on sensory evaluation of crude herbs and decoction pieces, involving subjective analysis of morphological traits (shape, color, texture), fracture surfaces, and organoleptic properties (odor, taste), with a focus on form, color, odor, and taste. This approach derives from the concept of Daodi authenticity—a TCM-specific quality criterion for medicinal materials cultivated in optimal geographic regions through centuries of empirical validation. Despite lacking quantitative rigor, the standards of Daodi remain foundational in pharmacopeial evaluations (e.g., Chinese Pharmacopoeia) and retain practical significance in regulatory practice [11].

Modern TCM quality regulation began with physicochemical analytical methods introduced in the *1953 Chinese Pharmacopoeia*. Regulatory models progressively adopted quality control frameworks of chemical drugs and natural medicines, employing spectroscopic and chromatographic techniques to quantify active constituents. While this chemical-centric quality control paradigm remains central to current standards, emerging methods such as chemo-biological fingerprinting and bioactivity assessment are gaining traction [12].

The establishment of TCM registration standards evolved alongside advancements in safety, efficacy, and quality control technologies. Key legislative milestones include the *Drug Administration Law of the People's Republic of China* (1985*)*, the *New Drug Approval Measures* (1985), the *Supplementary Provisions and Explanations on TCM-related Issues in the New Drug Approval Measures* (1987) and the *Regulations for the Administration of Drug Registration* (2002). These policies institutionalized modern drug evaluation criteria (quality, efficacy, safety), promoted research and development (R&D) innovation, and established centralized regulatory review systems [13, 14].

The scientific TCM regulation phase, initiated under China’s 2019 Drug Regulatory Science Action Plan, employs a tripartite framework integrating regulatory science bases, key laboratories, and research projects. This strategy aims to accelerate the research and application of new regulatory tools, standards, and approaches; develop TCM-specific regulatory tools and technical evaluation systems; enhance quality control by integrating modern technologies and traditional methods; strengthen clinical trial standards, thus promoting sustainable innovation [9, 15, 16].

### From traditional medicine to modern medicine: challenges and regulatory difficulties

#### The uniqueness of TCM theory

TCM operates within a distinct theoretical framework grounded in holistic principles and syndrome differentiation. This contrasts sharply with modern medicine's reductionist, evidence-based paradigm. TCM prioritizes individual constitutional and symptomatic variations, acknowledging differential patient responses to herbal therapies based on tolerance and sensitivity thresholds.

TCM clinical expertise derives from centuries of empirical practice, encompassing regional medicinal customs and folk remedies. Foundational texts like *Shang Han Lun (Treatise on Cold Damage Disorders)* and *Ben Cao Gang Mu (Compendium of Materia Medica)* codify ancient medical wisdom. The generational transmission of herbal therapies has demonstrated efficacy in managing region-specific ailments, while seasoned practitioners exhibit refined mastery of dosage customization and herb compatibility—core manifestations of TCM’s experiential legacy.

Clinical research confirms the therapeutic value of traditional compound formulations in alleviating symptoms and moderating chronic conditions such as diabetes and cardiovascular diseases [17, 18]. From a pharmaceutical development perspective, this accumulated human experience reduces exploratory research phases by providing preliminary validation of safety and efficacy profiles.

#### Complexity of TCM production and processing

TCM formulations predominantly originate from natural sources (plants, animals, minerals), with quality variability introduced at every stage. During cultivation, factors such as soil, climate, fertilization, and pest control can influence the growth and accumulation of active constituents in medicinal plants. Differences in processing methods and parameters during decoction and preparation can alter the pharmacological properties and constituent content. Additionally, production equipment, process controls, and storage/transportation conditions can further affect the stability and quality of TCM products.

The inherent chemical complexity of TCM, often comprising undefined constituents, is compounded by the clinical use of multi-herb formulations. Intricate manufacturing processes and constituent interactions complicate quality control and mechanistic studies. Current single-component analytical methods inadequately capture holistic quality attributes, failing to identify comprehensive pharmacodynamic components or toxicity sources, thereby undermining product consistency.

TCM’s network-based mechanisms, characterized by multi-target modulation rather than linear pathways, defy conventional reductionist analysis. Benefit-risk assessments face inherent challenges due to unclear endpoints and unpredictable interactions, hindering the identification of core therapeutic actions or adverse reaction mechanisms. Existing evaluation frameworks struggle to precisely predict outcomes across diverse populations, often falling short of modern regulatory demands [19, 20].

#### Adaptability of TCM regulatory frameworks

The inherent quality variability of TCM materials is influenced by multiple factors, including geographical origin, harvest timing, and processing techniques. These variations in active ingredient content across production regions pose challenges to the batch consistency in clinically derived TCM products. Further obstacles include declining expertise in traditional processing methods and insufficient precision in industrial parameter control, hindering quality stability and market access.

Traditional clinical documentation, often comprising anecdotal case reports or oral histories, lacks standardized data collection per modern trial requirements (e.g., defined inclusion criteria, dosing precision, and follow-up protocols). Translating fragmented empirical knowledge into regulatory-compliant evidence for drug approval remains a significant barrier. Current clinical trial designs, employing fixed dosages and uniform protocols, inadequately address individual variability, yielding risk–benefit assessments that poorly reflect real-world TCM applications across diverse populations. This limitation restricts the precise application and promotion of TCM [21].

While clinical experience provides preliminary safety insights, systematic monitoring of rare adverse events, long-term toxicity, and drug interactions remains absent. Modern regulations mandate comprehensive risk assessments, particularly for combination therapies. However, mechanistic uncertainties regarding interactions between TCM's complex components and Western pharmaceuticals complicate safety evaluations, impeding integration into mainstream healthcare. The evaluation of the TCM efficacy and safety frequently diverges frommodern scientific standards [22].

**Fig. 2:**
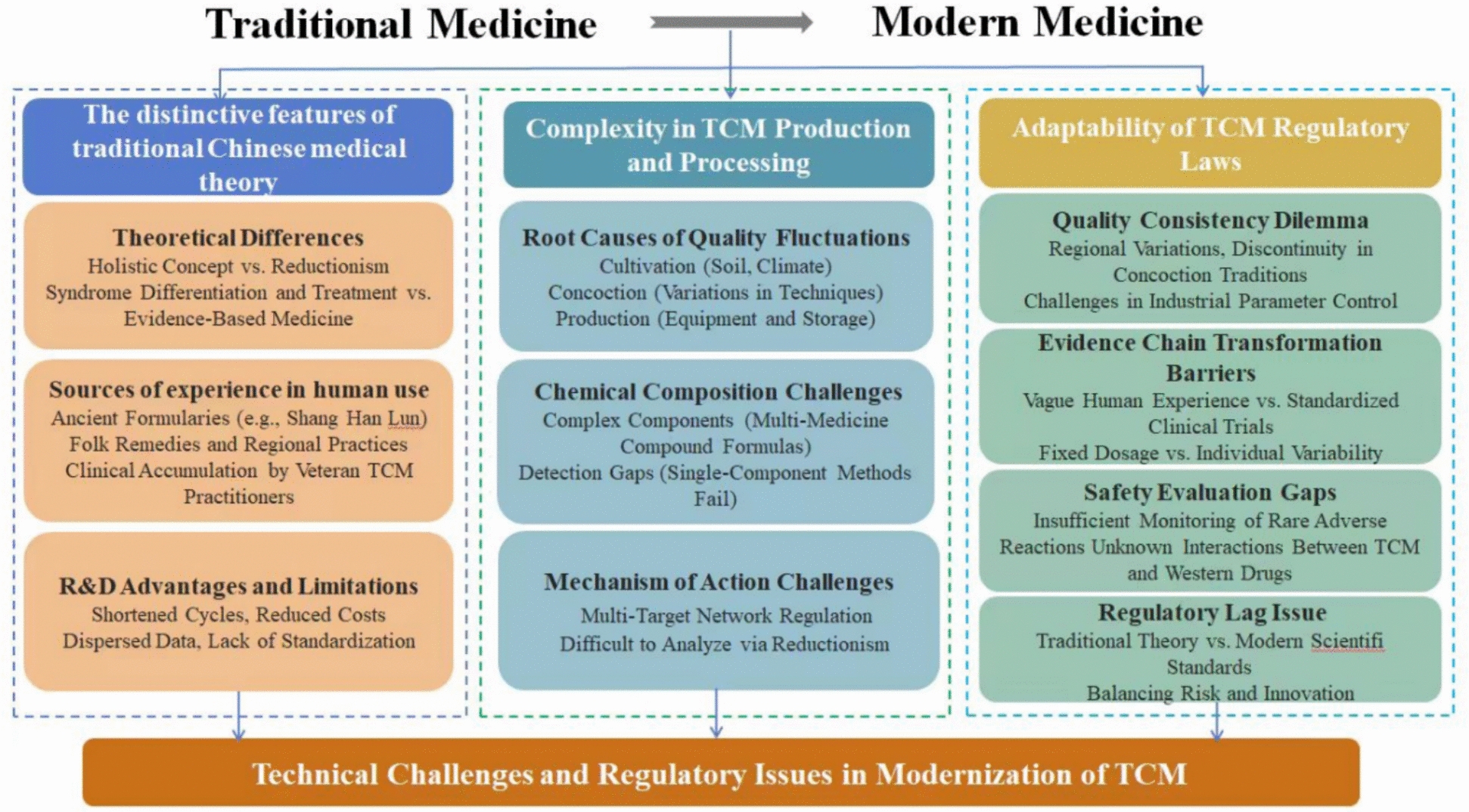
Technical challenges and regulatory issues in TCM modernization

Existing regulatory frameworks and standards, rooted in conventional drug evaluation, xhibit limited adaptability to TCM. Guidelines are lacking for translating novel analytical tools into regulatory decisions. Regulatory authorities face challenges in balancing risk and innovation while ensuring public health. Key issues that urgently need to be addressed include the formulation of reasonable review standards, the regulation of new tool applications, and the integration of modern scientific technologies and methods into the evaluation and regulation of TCMs, all while respecting TCM theories (Fig. 2).

### Scientific implications and significance of TCMRS

#### The proposition and scientific implications of TCMRS

Regulatory science, initially a public management tool for mitigating objective risks by proposing new tools, methods, and models, has evolved into a strategic discipline driving global drug innovation promoted by major global drug regulatory agencies. It has significantly advanced the synchronized research, registration, and evaluation of innovative drugs worldwide [9]. The formation and development of TCMRS have distinct Chinese characteristics, shaped under China’s Scientific Development Concept. This journey began with the emergence of drug review system reforms, followed by China’s Drug Regulatory Science Action Plan, the establishment of national key laboratories, and the integration of TCM-specific regulatory innovations [16].

The term regulatory science emerged in 1970. By 2010, the FDA formally defined drug regulatory science, and by 2019, TCMRS was first officially referenced in China’s Drug Regulatory Science Action Plan [4, 8]. Compared to well-established regulatory frameworks for chemical drugs, biologics, and medical devices, TCMRS has a shorter history of international research and remains less extensively studied. In 2023, we defined TCMRS as an emerging interdisciplinary field rooted in the dual attributes of Chinese medicine as both therapeutic practices and regulated drug products [9]. By interdisciplinary knowledge and technology integration of TCM, Western medicine, and regulatory science, TCMRS develops tailored tools, standards, and approaches to assess the safety, efficacy, quality, and benefit-risk profiles of Chinese medicinal products, including crude herbs, decoction pieces, and patent formulations [9]. TCMRS integrates traditional quality control methods, such as sensory evaluation of morphological traits (e.g., appearance, color, odor) and physicochemical constituent analysis, with modern technologies like bioassays and chromatographic fingerprinting. This hybrid approach ensures comprehensive quality monitoring of Chinese medicinal products.

**Fig. 3:**
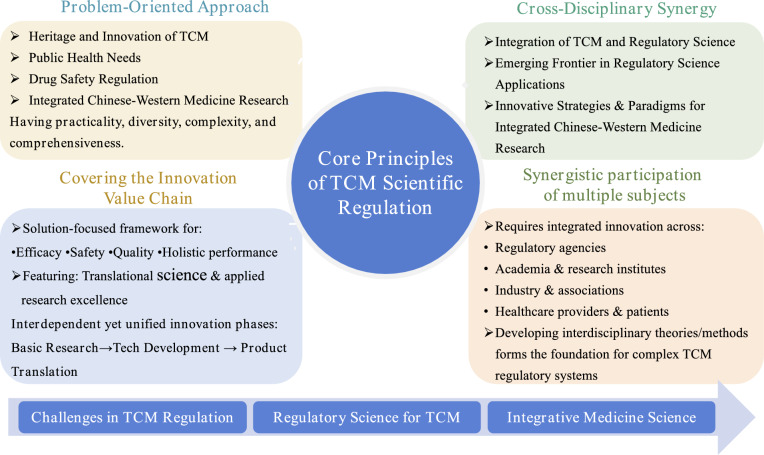
Scientific connotation of TCM regulatory science

TCMRS exhibits four distinctive features: (1) Problem Orientation, (2) Multidisciplinary Convergence, (3) Innovation Value Chain, and (4) Multi-Stakeholder Collaboration (Fig. 3).

(1) Problem Orientation: Distinct from discipline-oriented scientific research, TCMRS, as an emerging frontier discipline, exhibits typical characteristics of post-academic science, which is closely integrated with industry and the economy, referring to scientific research driven by by the values of pursuing productivity and material wealth. Functioning as a novel convergent scientific paradigm, TCMRS is dedicated to addressing critical socioeconomic challenges, including the inheritance and innovation of TCM, public health demands, pharmaceutical industry development, and drug safety regulation. These issues reflect the practical, multifaceted, complex, and integrative nature of TCMRS in bridging Chinese and Western medical research. By distilling major scientific and technological challenges within TCM regulation, foundational research and technological innovation can yield new regulatory tools, standards, and approaches. This advancement facilitates the establishment of a TCM-specific technical framework for evaluation and approval systems, thereby advancing the national strategy of high-level TCM inheritance and innovation. (2) Multidisciplinary Convergence: Diverging from traditional single-disciplinary, interdisciplinary, or cross-disciplinary research paradigms, TCMRS represents both an emerging application of regulatory science in TCM oversight and a novel strategy for convergence research integrating Chinese and Western medicine. Its research focus arises from real-world challenges that transcend conventional disciplinary knowledge, necessitating the dismantling of academic silos and the deep integration of cross-disciplinary methods, tools, paradigms, and concepts. While promoting multidisciplinary integration, TCMRS does not negate disciplinary specialization; instead, it recognizes that disciplinary depth remains indispensable for solving specific problems. This dual emphasis underscores the complementary roles of disciplinary development and convergence, highlighting the importance of both academic infrastructure and talent cultivation. (3) Innovation Value Chain: In traditional research models, the front-end basic research (often led by academia) and the back-end technological application and product development (driven by industry) lack effective connectivity. TCMRS, however, emphasizes solving critical drug-related issues, such as efficacy, safety, quality, and comprehensive performance evaluation, embodying the attributes of strong translational and applied science. Its emergence aligns closely with advancements in translational medicine. Notably, TCM’s millennia-old developmental model of “clinical practice → theoretical refinement → clinical practice” resonates with modern translational science. Both paradigms share the goals to accelerate innovative drug development and exhibit overlapping phased characteristics in implementation, where foundational research, technological development, and product translation operate as distinct yet unified stages. (4) Multi-Stakeholder Collaboration: The advancement of TCMRS requires resource integration and collaborative innovation among regulatory agencies, universities, research institutions, industries, professional associations, healthcare providers, and patients to develop its multidisciplinary theories and methodologies. Regulatory authorities serve as initiators, advocates, and practitioners of regulatory science, while universities, associations, industries, and patients act as participants and stakeholders. A synergistic network involving government, industry, academia, and research entities, along with partnerships supporting the translation of scientific progress into tangible products, forms the foundation for addressing the complexity of TCMRS [23–25]. This multi-stakeholder coordination ensures the integration of diverse expertise and resources essential for systemic progress.

#### The importance of TCMRS as an emerging discipline

Contemporary drug regulatory science is transitioning toward lifecycle management, technology-driven innovation, and global collaboration while balancing rapid technological advancements with regulatory stability. The integration of regulatory and interdisciplinary sciences into TCMRS aligns with the dual mandates of China’s National Medical Products Administration (NMPA): ensuring drug safety and fostering TCM’s heritage and innovation. This shift reflects proactive adaptation to evolving challenges in TCM orthodoxy, technological progress, industrial growth, public health demands, and international competition, underscoring TCMRS’s significance as an emerging discipline [24].

First, TCMRS extends regulatory science into novel domains. Conventional regulatory frameworks focus predominantly on chemical drugs and biologics, neglecting TCM’s unique complexities, such as its holistic theories, herbal formulations, and quality control challenges (e.g., raw herbs, extracts, and patent medicines). Existing tools are insufficient for TCM’s regulatory needs, necessitating a discipline that transcends traditional boundaries. Beyond its focus on evolutionary science and boundary science—such as hypothetical science, evidence-based science, partially reproducible science, scientific speculation, and scientific judgment—it places greater emphasis on the integration of TCM, life sciences, management science, and engineering. In other words, TCMRS is an evolving, boundary, and fusion science that remains under development, with areas that have not yet been scientifically validated or universally accepted. Crucially, it seeks to establish TCM-specific evaluation systems, avoiding the pitfalls of imposing Western standards on TCM through context-sensitive scientific validation.

Second, TCMRS addresses the incongruity of Western-style regulation of TCM. As chronic and complex diseases (e.g., metabolic disorders, neurodegenerative diseases, malignant tumors) challenge single-target drug paradigms, multi-target therapies gain prominence. The unique characteristics of TCM—including its non-antagonistic holistic regulation, safety profiles grounded in graded pharmacological properties and low toxicity, and quality control methods combining sensory evaluation with biological assessment—have gained substantial academic recognition. Notably, core concepts such as the authentic herbs theory (emphasizing geographical specificity in herb quality), toxicity-reduction processing techniques for herbal slices, and Yi Yin’s decoction theory for compound formulations underscore the interconnected quality-efficacy continuum from raw materials to processed products and finalized prescriptions. Previously underappreciated holistic pharmacological mechanisms and clinical values of herbal formulas are now being systematically re-evaluated. While comparative analyses of disease-targeted biomolecular frameworks in Western medicine and TCM’s holistic regulatory principles persist, academic consensus remains elusive regarding traditional medicine’s unique mechanisms and therapeutic advantages. TCMRS emerges as a critical interface for reconciling these paradigms, countering the limitations of Western-centric regulatory frameworks while operationalizing China’s scientific development strategy in healthcare. Strategically, TCMRS prioritizes establishing safety and efficacy assessment protocols tailored to traditional medicine’s characteristics, expediting evidence-based systems that harmonize TCM theory, clinical practice, and empirical data to objectively evaluate risk–benefit profiles. In this process, the development of fusion science and transformational science provides a rapid pathway for TCMRS, forming its core content and key areas of translational research.

Third, TCMRS facilitates the integration of Chinese and Western medical knowledge systems. Though nascent, this discipline is evolving into a vital framework for evaluating traditional medicines’ efficacy, safety, quality control, and risk–benefit assessment, serving as an innovative paradigm in interdisciplinary research. The absence of a unified knowledge system, stemming from divergent theoretical foundations between TCM and biomedicine, poses challenges in developing traditional medicine-specific evaluation protocols. By synthesizing natural sciences, social sciences, and humanities, TCMRS aims to achieve end-to-end quality control, streamline regulatory processes, and foster global regulatory harmonization through fusion science methodologies. A landmark development occurred in October 2020 when NMPA partnered with the Chinese Academy of Medical Sciences to standardize clinical trial evaluation criteria, explore tiered management of research centers, and restructure clinical trial infrastructure [26]. Leveraging data transparency and shared regulatory science resources, this initiative catalyzes a paradigm shift in TCM research ecosystems, systematically enhancing clinical trial rigor and pharmacovigilance capabilities.

## Progress and research highlights in TCMRS

### Regulatory classification reform for TCM new drugs redefines innovation pathways

In September 2020, the NMPA introduced the *Requirements for Registration Classification and Application Dossiers of TCMs*, instituting a novel four-tier classification system for TCM pharmaceuticals. This framework, tailored to TCM's R&D patterns and clinical utility, categorizes new TCM drugs as: (1) innovative TCMs, (2) modified new drugs of TCMs, (3) compound preparations of TCMs originated from classic recipes, and (4) TCMs with identical name and identical formulations (Table 1).

**Table 1:** TCM registration categories and documentation requirements (September 2020)

Category	Definition	Subcategories	Remarks
1, innovative TCMs	Refers to new TCM formulations with clinical value that are not included in the National Drug Standards, Drug Registration Standards, or the *Catalogue of Ancient Classic Prescriptions* issued by the National Administration of Traditional Chinese Medicine, and are not marketed outside China	1.1 TCM Compound Preparations 1.2 Extracts and Preparations Derived from Single Plant Sources 1.3 New Medicinal Materials and Their Preparations	Emphasizes novelty in therapeutic efficacy, prioritizes clinical value assessment, and addresses unmet clinical needs
2, modified new drugs of TCMs	Refers to modifications of marketed TCM drugs, including changes in administration routes, dosage forms, or expanded indications, that demonstrate clinical advantages	2.1 Preparations with Altered Administration Routes 2.2 Preparations with Altered Dosage Forms 2.3 TCM Drugs with Expanded Indications 2.4 Preparations with Significant Changes in Pharmaceutical Substance Basis or Drug Absorption/Utilization Due to Manufacturing Process or Excipient Modifications	“Expanded Indications” require submission as a new drug application instead of a supplemental application Significant changes in pharmaceutical substance basis or absorption/utilization due to manufacturing process modifications are regulated as modified new drugs Encourages in-depth research and secondary development of marketed TCM drugs to enhance quality and clinical value
3, compound preparations of TCMs originated from classic recipes	Refers to formulations documented in ancient TCM classics that comply with the *Traditional Chinese Medicine Law of the People's Republic of China*, remain widely used, and demonstrate proven efficacy with distinct therapeutic characteristics	3.1 Preparations Managed Under the Catalog of Ancient Classical Formulas 3.2 Other Preparations Derived from Ancient Classical Formulas	Both subcategories must adhere to traditional processing methods and traditional routes of administration, and describe functions/indications in TCM terminology •Subcategory 3.1 requires pharmaceutical and non-clinical safety studies •Subcategory 3.2 additionally mandates systematic summarization of human use experience and evaluation of clinical value
4, TCMs with identical names and formulations	Refers to preparations with identical generic names, formulas, dosage forms, functions/indications, usage, and daily herbal dosage to a marketed TCM drug while ensuring non-inferiority in safety, efficacy, and quality controllability		Not equivalent to generic drugs; approval depends on comparative study results with the marketed drug, not merely quality standard consistency Requires non-inferiority in safety, efficacy, and quality controllability under identical generic names, formulas, dosage forms, functions/indications, usage, and daily herbal dosage

This revision follows an in-depth evaluation of historical TCM review practices and integrates advancements from China’s broader drug regulatory reforms. By aligning with TCM’s unique R&D paradigms, the policy represents a transformative advancement in pharmaceutical policy. A key innovation lies in its recognition of TCM’s developmental patterns and prioritization of TCM-specific attributes. Departing from earlier classification systems that emphasized chemical composition criteria, the revised framework adopts a clinical value-driven approach. It optimizes registration standards by prioritizing therapeutic innovation, pharmacological uniqueness, and regulatory feasibility, thereby incentivizing clinically meaningful TCM development.

Innovative TCMs target unmet medical needs, while modified new drugs of TCMs must demonstrate enhanced efficacy or safety profiles. Unlike prior systems that had narrowly focused on isolated active ingredients, this reform establishes a globally relevant framework for evaluating compound-based TCM innovations [27, 28], emphasizing holistic therapeutic value.

### The “three-integrated” evidence evaluation system for TCM efficacy evaluation

In both preclinical and clinical research contexts, the development of novel methodologies for TCM requires alignment with its distinctive therapeutic paradigms. As the material basis for TCM’s historical continuity and modernization, its evolution spans from empirical documentation in Shennong Bencaojing to Yiyin’s decoction formulations, progressing from single-herb applications to synergistic combination therapies. Compound decoctions and their derivatives—grounded in TCM theory and clinical validation—epitomize the discipline’s core therapeutic strategies. Guided by TCM’s diagnostic framework (Li Fa Fang Yao: theory, pattern, formula, and drug) and compounding principles (Jun Chen Zuo Shi: sovereign, minister, assistant, servant), personalized combination therapies operationalize TCM’s moderation-integrated-balance presupposition [29] and its unique pharmacodynamic mechanisms under the Yiyin Decoction Theory [30].

TCMRS ensures methodological robustness in clinical trial design for traditional medicines. The innovative “three-integrated” evidence framework integrates empirical knowledge from TCM’s extensive clinical legacy into regulatory evaluations. TCM’s practice-oriented epistemology, exemplified by its accumulated real-world data and experiential insights, forms the foundation for evidence guidelines such as *Guidance Principles for Communication under the “Three-Integrated” Registration Review Evidence System (Trial)* and *Guidelines for Clinical Development of New TCM Compound Formulation Drugs Based on Human Use Experience (Trial)*. These protocols acknowledge TCM’s R&D trajectories, establishing novel pathways for regulatory review while expanding opportunities for clinical pharmacists in drug development. Retrospective analysis of clinical cases enables the identification of disease-specific therapeutic patterns, informing both drug innovation and clinical practice. By employing advanced data science methodologies, TCMRS fosters an evidence system synthesizing theoretical principles, real-world evidence, and clinical trial data, while pioneering real-world evidence frameworks for TCM [31–33]. Real-world studies (RWS) demand critical evaluation of inherent limitations, necessitating methodological innovations to strengthen validity, reliability, and bias mitigation [34].

Randomized, double-blind, placebo-controlled clinical trials require rigorously designed experimental and control groups to evaluate TCM efficacy. Patient stratification based on TCM syndrome differentiation should further refine study cohorts, ensuring alignment with TCM’s therapeutic principles. The selection of efficacy endpoints must reflect TCM’s clinical strengths, prioritizing outcomes such as syndrome severity scores and quality-of-life metrics. TCMRS advocates integrating these TCM-specific measures with modern biomedical biomarkers (e.g., imaging or laboratory parameters) to ensure comprehensive therapeutic effect evaluation. Innovations in trial design, including real-world evidence, adaptive/enrichment strategies, patient-centric endpoints, and biomarker-driven surrogate outcomes, are strongly encouraged to advance TCM drug development [35].

Preclinical pharmacological studies must adhere to TCM theory, employing experimental models that mirror its clinical applications and mechanistic uniqueness. These studies shall comply with the principle of “tailoring analytical approaches to address specific research questions”, ensuring scientifically rigorous experimental designs and standardized protocols to generate objective and reproducible data. TCM pharmacological trials must follow core principles of randomization, controlled conditions, and replicability. The integration of emerging technologies to investigate TCM’s mechanisms of action is strongly encouraged, incorporating insights from international drug development frameworks. When novel methodologies or technologies are implemented in TCM pharmacological studies, rigorous validation of these methodologies is essential to confirm their reliability [35].

### Enhanced “full-process” safety concept in TCM safety evaluation

The TCM safety evaluation forms a holistic framework covering all stages from drug discovery through clinical application to market approval. This evidence-driven paradigm systematically conducts toxicity assessments through a sequential “clinical-non-clinical-clinical-post-marketing reevaluation” approach, embedding safety monitoring throughout the drug’s lifecycle. Effective risk management for TCM safety must address medicinal material cultivation and traceability, processing/manufacturing techniques, production standardization, and quality control. Furthermore, it necessitates systematic toxicological studies, risk-mitigated clinical trials, and post-marketing surveillance to ensure safety compliance [36].

TCMRS employs modern toxicological methodologies to assess toxicity and safety profiles holistically. This includes characterizing toxicological patterns, establishing dose–response relationships, elucidating mechanisms of action, and identifying target organ risks. A robust adverse reaction monitoring system enables the collection and analysis of clinical safety data, facilitating early detection of potential hazards. Advanced analytics, such as big data mining, can uncover adverse reaction trends linked to TCM usage, informing label updates or dosage adjustments to mitigate risks. Additionally, medicinal herbs and preparations are susceptible to contamination by exogenous pollutants (e.g., pesticide residues, heavy metals) during cultivation, processing, or storage. Regulatory frameworks utilize cutting-edge detection technologies—including high-performance liquid chromatography-mass spectrometry (HPLC–MS) and inductively coupled plasma mass spectrometry (ICP-MS)—to precisely identify and quantify contaminants, ensuring TCM product safety [35].

Given the unique characteristics of TCM, such as multi-component complexity, batch-to-batch variability, and incomplete understanding of composite toxicity mechanisms, studies have explored the application of Microtox technology in TCM toxicity evaluation. These investigations focus on methodologies, pathways, and applications to advance the development of a comprehensive toxicity assessment framework, including toxicity grading and compatibility-based detoxification strategies [37, 38].

To address clinical safety challenges in TCM, researchers have established a four-dimensional collaborative platform for pharmacovigilance. This framework integrates risk signal detection, evidence consolidation, risk–benefit analysis, and risk communication, thereby facilitating the translation of TCM pharmacovigilance theories into practical applications [39].

### Systematic quality control in TCM

While debates persist regarding approaches for evaluating the safety and quality of TCM compounds, internationally recognized principles for herbal and natural drug products have been established [30]. These principles encompass raw material quality assurance, production process monitoring, and the development of robust quality standards. Scientifically validated standards are critical for ensuring TCM quality. Guided by drug lifecycle management, TCMRS utilizes multidisciplinary methodologies, including chemical and biological analyses, to establish quality control systems that holistically assess TCM quality. This framework integrates comprehensive and stage-specific control measures, ensuring rigorous management of all product lifecycle stages affecting quality, from sourcing and production to stability testing and post-market surveillance.

Quality standard research prioritizes aligning control indicators with TCM safety and efficacy, developing evaluation frameworks tailored to TCM’s unique characteristics and registration categories. Key production control points include raw material authentication, emphasizing geographical origin and botanical authenticity. As production processes critically influence pharmacological outcomes, stringent process controls are essential. Quality control phases prioritize comprehensive standards, including efficacy-linked indicators (e.g., extract quantification, chromatographic fingerprints, multi-component assays) and rigorous safety parameters. Stability studies require selecting markers that reflect real-world storage and usage conditions [39, 40]. Recent advancements include standard decoctions, with defined attributes and preparation methods, serving as benchmarks for quality control across development and post-market evaluation [41].

The identification of quality markers (Q-Markers) or biomarkers in TCM requires integrating chemical components, bioactivity, and therapeutic efficacy through multidimensional models to advance scientifically robust methods for evaluating TCM quality. By aligning quality parameters with regulatory science, systematic traceability studies throughout the product lifecycle, from raw material cultivation to clinical outcomes, may establish a regulatory framework to ensure consistency in TCM formulations [42].

In December 2020, the Center for Drug Evaluation (CDE) under NMPA introduced the *Technical Guidelines for Bioactivity-Based Testing of Traditional Chinese Medicine (Trial)*. These guidelines directly link bioactivity to TCM safety and efficacy, capturing the multi-component, holistic nature of TCM through measurable biological responses. This approach constitutes a proactive and exploratory strategy for quality control, offering substantial value for advancing innovation in TCM drug development and fostering high-quality industry growth [43]. Microtox technology enables a novel rapid detection method applicable to toxicity screening and bioactivity testing for TCM injections, enhancing the reliability of quality control and safety risk monitoring [44].

In conclusion, while preserving TCM’s personalized combination therapies, clinical value, and theoretical foundations, its formulations demonstrate defined consistency in composition and proportion, clarity in chemical constituents, stable efficacy, and synergistic therapeutic effects—aligning with modern pharmacological principles. Central to TCMRS is the advancement of novel tools, methodologies, and standards tailored to its unique characteristics, aiming to holistically evaluate product risks and benefits through efficacy, safety, and quality control frameworks. Furthermore, benefit-risk assessments incorporate integrated pharmacoeconomic and societal impact analyses across the drug lifecycle, serving as pivotal considerations in clinical research, market authorization, and post-market regulatory decisions. Given the challenges of comparing heterogeneous metrics in benefit-risk evaluations, alongside TCM’s distinct holistic efficacy mechanisms (e.g., balanced regulation) and safety paradigms (e.g., graded toxicity and compatibility theories), innovative methodologies are essential to address uncertainties inherent in these assessments [45].

## Future perspectives: strategic directions and prospects for TCMRS

### Strategic goals and key priorities in TCMRS

Globally, the FDA stands as a preeminent regulatory authority, with its drug regulatory framework and pharmaceutical industry serving as international benchmarks. The FDA’s regulatory excellence stems from its robust internal scientific rigor and culture, augmented by external professionalism that reinforces its authoritative reputation [46–48].

**Fig. 4:**
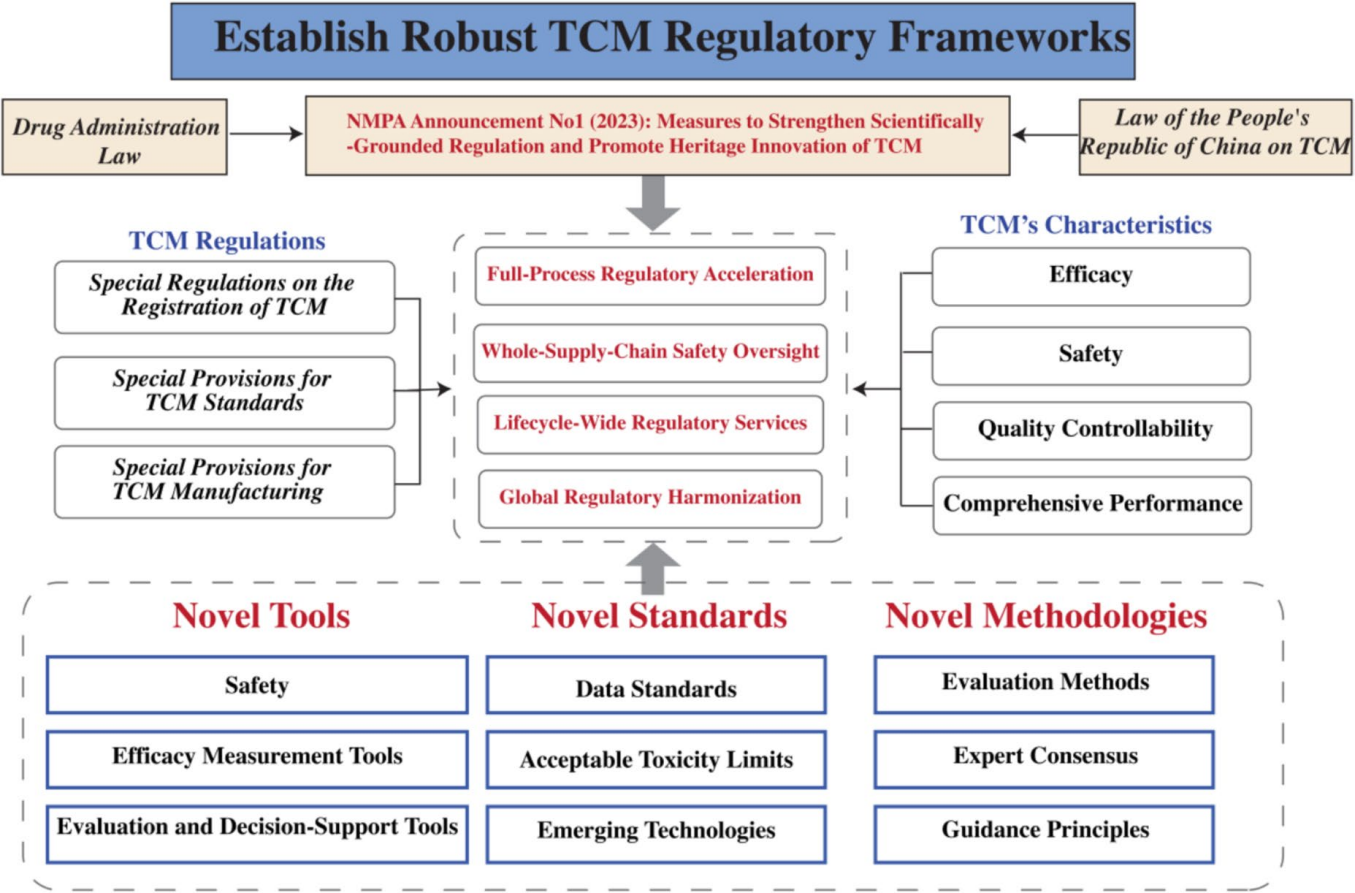
From regulatory science to scientific regulation: construction of the TCM scientific regulatory system

TCM, with its dual role as both a therapeutic practice and a product embedded within scientific, cultural, economic, and ecological contexts, introduces unique regulatory complexities. These inherent characteristics position TCMRS as an interdisciplinary and emerging field. Its development is closely tied to advancements in modern regulatory science and translational science, which provide foundational theories, methodologies, and frameworks for innovation. Recent progress in TCMRS includes reforms in evaluation and approval processes for new drugs, optimization of standardization mechanisms, enhanced supply chain safety monitoring, translational research frameworks, and international regulatory harmonization. These achievements have significantly contributed to establishing robust TCM regulatory frameworks, advancing lifecycle-based oversight, industry-wide safety protocols, and global regulatory cooperation (Fig. 4) [9, 16, 24, 35].

The strategic trajectory of TCMRS centers on holistic regulatory innovation. This entails reinforcing end-to-end evaluation and approval mechanisms, strengthening supply chain safety oversight, delivering lifecycle-based regulatory services, and advancing international regulatory collaboration. The overarching objective is to establish a globally pioneering TCM regulatory framework that embodies Chinese regulatory principles, accommodates TCM’s distinctive attributes, and ensures rigorous safety standards alongside sustainable industrial growth.

**Fig. 5:**
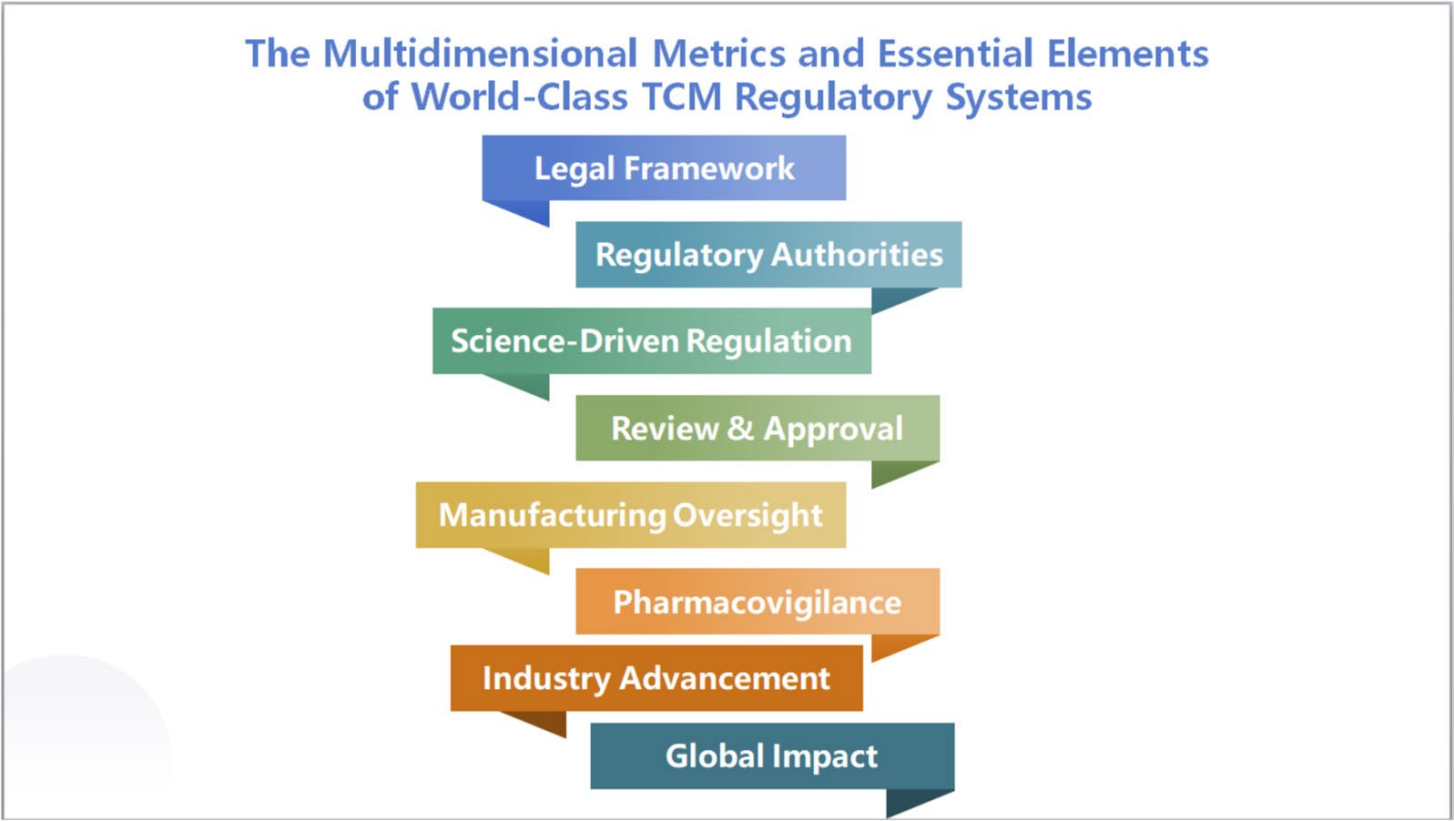
Multi-dimensional Evaluation System and Core Components of the TCM Excellence Regulatory Framework

Developing an exemplary TCMRS constitutes a pivotal mandate for contemporary drug regulatory authorities. The system must be structured hierarchically, prioritizing Chinese regulatory identity, TCM-specific paradigms, and global leadership. It should integrate multidimensional elements, including institutional frameworks, methodological rigor, evaluation protocols, production surveillance, pharmacovigilance systems, industry advancement strategies, and global regulatory harmonization (Fig. 5). To realize this vision, TCMRS must tackle critical challenges tied to building an advanced regulatory system. This necessitates strategic prioritization of focus areas, challenges, and initiatives, encompassing targeted domains, high-priority actions, phased milestones, and implementation strategies, to optimize resource allocation, mitigate risks, and systematically advance regulatory objectives [49].

### Emerging technologies accelerating the advancement of TCMRS

TCMRS integrates multidisciplinary knowledge spanning pharmacology, medicine, biology, chemistry, informatics, and jurisprudence, with a focus on quality assurance and risk management across the TCM lifecycle. During the 13th Five-Year Plan period, two TCMRS research bases were established in collaboration with the China Academy of Chinese Medical Sciences and Beijing University of Chinese Medicine. Concurrently, 27 national key laboratories dedicated to TCM regulation were developed in partnership with drug testing institutions, academic institutions, and research organizations. These initiatives facilitated the timely translation of novel regulatory tools, standards, and methodologies into practice, ensuring alignment with pharmaceutical technological advancements. From raw material cultivation and processing to formulation development, clinical trials, market authorization, and post-marketing surveillance, including adverse drug reaction monitoring, TCMRS leverages advanced technologies and scientific principles to establish regulatory guidelines and technical standards tailored to traditional drug attributes. This framework has driven regulated growth of the TCM industry and bolstered its global competitiveness [16, 49]. Amidst rapid technological advancement, the deep integration of TCM and technology is increasingly becoming a prominent trend. For example, AI technology can rapidly conduct deep mining of vast amounts of classical Chinese medicine literature, providing an endless source of inspiration for new drug research and development. Simultaneously, modern scientific methods enable in-depth analysis of the components and pharmacology of TCM, driving the deeper integration of TCM with modern medicine, and helping Chinese medicine gain wider recognition on the international stage. Moving forward, emerging technologies are poised to further catalyze TCMRS innovation, creating strategic opportunities for transformative progress in the field.

#### Integration of multi-omics technologies

The integration of multi-omics technologies, including genomics, transcriptomics, proteomics, and metabolomics, facilitates a holistic analysis of traditional drugs’ mechanisms of action and material foundations, yielding comprehensive insights for benefit-risk assessments. By synthesizing multi-omics datasets, systems biology models of TCM can be developed to predict safety and efficacy with greater precision [50–52]. Network pharmacology aggregates extensive datasets encompassing drug targets, disease-associated genes, and protein–protein interactions, constructing drug-target-disease networks. This approach enables the systematic elucidation of how multicomponent TCM formulations synergistically interact with complex disease pathways. Furthermore, critical therapeutic targets can be identified through network topological attributes, predicting potential off-target interactions and adverse reaction mechanisms. This optimization of formulation composition enhances the benefit-risk profile of TCM [53, 54]. Luqi Huang et al. employed an integrative pharmacology approach, focusing on dual therapeutic pathways of antiviral activity and anti-inflammatory effects, to systematically investigate the molecular targets and mechanisms of active ingredients in Huashi Baidu decoction against COVID-19 [55]. Jun Yu et al. explored the effect of Pien Tze Huang on colorectal cancer using multi-omics approaches. The study revealed that Pien Tze Huang improved gut barrier function, enhanced gut microbiota diversity, and suppressed oncogenic and pro-inflammatory pathways, thereby suppressing colorectal carcinogenesis [56]. Thus, multi-omics and network pharmacology, as emerging technologies, play a significant role in promoting the development of TCM.

#### Big data and artificial intelligence applications

Harnessing big data technologies, this methodology aggregates and synthesizes extensive datasets related to TCM and herbal remedies, encompassing clinical outcomes, adverse drug reactions (ADRs), and pharmacoeconomic metrics. Artificial intelligence (AI) algorithms are employed to mine and analyze these datasets, revealing latent patterns and correlations to deliver intelligent decision-support tools for risk–benefit evaluations. For example, machine learning (ML) algorithms can generate predictive models for TCM-related ADRs, enabling preemptive identification of safety risks. Such models allow regulators to contextualize drug profiles, streamline review timelines, and improve assessment accuracy. Advanced ML techniques—including support vector machines (SVMs), random forests, and deep neural networks—are utilized to construct toxicity prediction models. These models integrate chemical structures, physicochemical properties, and bioactivity data of known toxic agents (e.g., TCM monomers and extracts) with molecular fingerprints, lipophilicity, and acid–base characteristics to estimate toxicity probabilities for novel TCM components or formulations. This predictive capability informs early-stage R&D decisions and mitigates toxicity risks during preclinical and clinical trials [57]. Clinical big data, comprising patient demographics, symptomatology, laboratory findings, TCM syndrome assessments, and treatment regimens, are analyzed via ML algorithms to develop efficacy evaluation frameworks for TCM interventions [58]. Real-world evidence (RWE) leverages multisource datasets from electronic medical records (EMRs), healthcare insurance databases, and ADR monitoring systems to evaluate TCM utilization in naturalistic clinical settings. Large-sample cohort studies enable longitudinal tracking of TCM safety profiles and detection of rare but severe ADR signals. Concurrently, statistical methodologies such as propensity score matching facilitate comparative efficacy analyses between TCM and conventional therapies or distinct TCM regimens, quantifying net benefits across demographic, comorbidity, and geographic subgroups [59].

#### New technologies, novel dosage forms and risk assessment

The development of innovative dosage forms for traditional medicines, such as sustained-release formulations or nanocarrier-based systems, necessitates integrating regulatory science to evaluate pharmaceutical properties and clinical efficacy. For instance, nanocarrier technologies (e.g., nanocrystals) enhance the bioavailability of poorly soluble TCM compounds. However, their regulatory frameworks face challenges. Agencies such as the FDA and the EMA are establishing quality standards for nano-herbal products, including particle size distribution and surface properties. Nevertheless, the lack of harmonized guidelines impedes industrial scalability [60, 61]. Emerging delivery technologies, including microneedles, remain hindered by underdeveloped regulatory frameworks, underscoring the need for international collaboration to standardize protocols [62]. Plant-derived biologics (e.g., the anti-Ebola monoclonal antibody ZMapp®) follow distinct manufacturing and regulatory pathways compared to conventional drugs, requiring tailored guidelines to address unique attributes of plant expression systems, such as glycosylation patterns [63]. TCM safety evaluations should emphasize acute/chronic toxicity, allergenicity (e.g., skin sensitization risks from herbal extracts), and contraindications for vulnerable populations (e.g., pregnant individuals) [64]. Japan’s Kampo research has pioneered exploratory safety pharmacology to implement early-stage safety screening, thereby reducing late-phase attrition in drug development [65]. Furthermore, environmental risk assessments—such as ecotoxicity analyses of TCM production waste—are increasingly integrated into regulatory frameworks [66].

#### Cross-domain interdisciplinary integration

TCMRS intersects diverse disciplines, including Chinese and Western medicine, regulatory science, statistics, and computer science, posing significant challenges for interdisciplinary collaboration. Experts from distinct fields often differ in knowledge bases, research paradigms, and methodologies, creating barriers to cohesive innovation. For example, biologists and data scientists may conflict over experimental design or data interpretation, hindering the seamless adoption of novel tools and limiting multidisciplinary synergy [67]. Interdisciplinary collaboration requires project-driven initiatives and educational coordination. Driven by new-generation information technologies such as artificial intelligence, big data, and the Internet of Things, we advocate for “AI + X”, actively applying new methods, technologies, and approaches from different disciplines to focus on solving key problems in their fields. Breaking down disciplinary silos and fostering interdisciplinary collaboration are essential to advancing TCMRS. For example, Cho employed the deep-learning models VGGNet16, ResNet50, and MobileNet to identify toxic herbs, including *Sinomenium acutum*, *Astolochia manshuriensis*, and *Akebia quinata*. The results from MobileNet-TL demonstrated remarkable capability in distinguishing these three herbs, surpassing human-level identification accuracy [68].

#### Regulatory framework enhancement and international harmonization

The global integration of TCM demands strengthened international collaboration to advance regulatory frameworks. Regulatory strategies vary regionally: the FDA and EMA require stringent clinical evidence for herbal products, while Japan accelerates approvals for selected Kampo medicines based on historical use (the empirical basis principle). Regulatory science must prioritize international harmonization, for example, via frameworks like the International Council for Harmonisation (ICH) of Technical Requirements for Pharmaceuticals for Human Use, to reconcile quality standards while preserving traditional medical distinctiveness [69, 70]. Furthermore, the use of Laboratory Developed Tests in TCM genetic testing necessitates clearly defined regulatory oversight to prevent inconsistencies arising from overlapping institutional jurisdictions [71]. In summary, TCMRS must synthesize modern analytical technologies, global coordination mechanisms, and traditional knowledge. Adopting risk-based assessments, it addresses challenges such as quality variability and complex interactions while bridging innovative technologies with traditional practices. Proactive engagement in global standard-setting initiatives, sharing research findings internationally, and adopting advanced regulatory methodologies will amplify China’s leadership in TCMRS.

### Mechanisms of the TCMRS researcher alliance

The advancement of TCMRS has introduced innovative approaches and methodologies for regulating TCM and other traditional medicines. By applying novel tools, standards, and methodologies, TCMRS addresses regulatory challenges, enhances quality control, and ensures the safety and efficacy of public health interventions. Currently, TCMRS remains in an early development stage, with persistent gaps in technical applications, standardization, and methodological implementation. Mechanisms for translating research outcomes into practical solutions require further refinement through targeted research and real-world application.

In February 2024, the Traditional Chinese Medicine Regulatory Science Coalition (TCMRSC) was established through collaborative efforts of experts from governmental, industrial, academic, and research sectors in TCM innovation and regulation. Guided by principles of “equality, openness, collaboration, efficiency, and innovation”, this platform fosters interdisciplinary dialogue and knowledge exchange, serving as a catalyst for TCMRS development by providing intellectual and innovative support [72]. The TCMRSC conducts the following activities to accelerate disciplinary advancement and build an excellent regulatory system for TCM: (1) The TCMRSC facilitates thematic discussions on scientific topics, common challenges, critical concerns, and recent research achievements in regulatory science for TCM across the entire supply chain. (2) The TCMRSC invites relevant coalition members to collaborate and exchange academic insights on recent scientific advancements in TCM regulatory research. (3) The TCMRSC conducts collaborative research on the scientific regulation of TCM, publishes a series of evidence-based research reports, synthesizes perspectives from coalition members and experts, and formulates policy recommendations for submission to regulatory authorities. (4) The TCMRSC systematically collects, organizes, and submits information regarding scientific research activities, achievements, and analytical reports related to TCM regulation, delivering these comprehensive resources to coalition members, policy-making entities, and senior professionals across academic and industrial sectors. (5) The TCMRSC establishes a platform for training and communication on TCM scientific research achievements. This platform will provide coalition members with knowledge and training related to supervision, evaluation and approval of TCM. (6) The TCMRSC organizes exchanges and cooperation with international academic organizations, collaborating with overseas institutions to conduct academic exchange activities.

To date, the TCMRSC has organized three thematic symposia: TCMRSC 2024–1 (Tianjin), TCMRSC 2024–2 (Beijing), and TCMRSC 2024–3 (Chengdu). These forums addressed critical regulatory challenges, including substitutes for rare medicinal materials, extract-based raw materials, preventive disease treatment drug development, and clinical translation of syndrome-specific TCM formulations. These discussions have accelerated TCMRS research, streamlined market translation of novel TCM products, and elevated the scientific rigor of TCM regulation [73–75].

## Conclusion

Natural products and traditional medicines are pivotal to global public health and healthcare security. Traditional medical systems, such as TCM, Ayurveda, Korean traditional medicine, and Unani, have evolved into structured frameworks with regional and global applications [76]. Regulatory science for TCM and herbal medicines constitutes an interdisciplinary field dedicated to ensuring product quality, safety, and efficacy through evidence-based methodologies. The evolution of TCM regulatory science has profoundly transformed industry practices by introducing advanced analytical tools, standardized protocols, and innovative evaluation frameworks. These developments have substantially enhanced regulatory efficiency, enabling comprehensive quality control from raw material sourcing to production processes, clinical efficacy validation, and post-market safety monitoring. Such advancements have driven the modernization of TCM practices across multiple dimensions. As cutting-edge disciplines like life sciences and artificial intelligence advance, TCMRS will increasingly incorporate technological innovations to establish a sophisticated framework aligned with TCM’s unique characteristics, positioning it as a globally recognized healthcare system. Concurrently, international collaboration requires urgent prioritization to harmonize regulatory standards, enabling TCM’s systematic integration into global healthcare. In 2024, the NMPA issued “Special Provisions on the Management of Traditional Chinese Medicine Standards”. Then the National Administration of Traditional Chinese Medicine released the “Traditional Chinese Medicine Standardization Action Plan (2024–2026)”, which explicitly stated that 180 domestic standards and 30 international standards for TCM will be completed by the end of 2026 [77]. In terms of TCM resources, we will capitalize on the established overseas TCM centers to accelerate the implementation of the “New Era Shennong Tasting Herbs Initiative”, aiming to discover new medicinal plant resources and genuine medicinal materials worldwide. Totally, to promote TCM internationalization, sustained investment in regulatory research should be coupled with strengthened talent development, interagency coordination, and industry-academia-research partnerships. By fostering global cooperation and science-driven governance, the establishment of TCM regulation can transition from administrative decision-making to empirically validated practices. This transformation will ensure TCM’s safe and effective application within modern medical systems while preserving its traditional heritage, ultimately securing its role as a revitalized, globally accessible form of medicine.

## Data Availability

Not applicable.

## Abbreviations

TCMRS: Traditional Chinese Medicine Regulatory Science
TCM: Traditional Chinese Medicine
FDA: U.S. Food and Drug Administration
R&D: Research and development
NMPA: National Medical Products Administration
RWS: Real-world studies
HPLC–MS: High-performance liquid chromatography-mass spectrometry
ICP-MS: Inductively coupled plasma mass spectrometry
Q-Markers: Quality markers
CDE: Center for Drug Evaluation
ADRs: Adverse drug reactions
AI: Artificial intelligence
ML: Machine learning
SVMs: Support vector machines
RWE: Real-world evidence
EMRs: Electronic medical records
EMA: European Medicines Agency
ICH: International Council for Harmonisation
TCMRSC: Traditional Chinese Medicine Regulatory Science Coalition
